# Resting-state connectivity of the amygdala is altered following Pavlovian fear conditioning

**DOI:** 10.3389/fnhum.2012.00242

**Published:** 2012-08-23

**Authors:** Douglas H. Schultz, Nicholas L. Balderston, Fred J. Helmstetter

**Affiliations:** ^1^Department of Psychology, University of Wisconsin-MilwaukeeMilwaukee, WI, USA; ^2^Department of Neurology, Medical College of WisconsinWauwatosa, WI, USA

**Keywords:** amygdala, fear conditioning, fMRI, resting-state, functional connectivity

## Abstract

Neural plasticity in the amygdala is necessary for the acquisition and storage of memory in Pavlovian fear conditioning, but most neuroimaging studies have focused only on stimulus-evoked responses during the conditioning session. This study examined changes in the resting-state functional connectivity (RSFC) of the amygdala before and after Pavlovian fear conditioning, an emotional learning task. Behavioral results from the conditioning session revealed that participants learned normally and fMRI data recorded during learning identified a number of stimulus-evoked changes that were consistent with previous work. A direct comparison between the pre- and post-conditioning amygdala connectivity revealed a region of dorsal prefrontal cortex (PFC) in the superior frontal gyrus that showed a significant increase in connectivity following the conditioning session. A behavioral measure of explicit memory performance was positively correlated with the change in amygdala connectivity within a neighboring region in the superior frontal gyrus. Additionally, an implicit autonomic measure of conditioning was positively correlated with the change in connectivity between the amygdala and the anterior cingulate cortex (ACC). The resting-state data show that amygdala connectivity is altered following Pavlovian fear conditioning and that these changes are also related to behavioral outcomes. These alterations may reflect the operation of a consolidation process that strengthens neural connections to support memory after the learning event.

## Introduction

Learning and memory are supported by a variety of neurobiological processes that ultimately modify the structure and function of the nervous system. Many of these processes alter the efficacy of neural connections. The process of changing these neural connections begins during exposure to the events and relationships to be encoded and continues after this initial experience as long term memory is formed and stored (for review see Kandel, 2001; Squire and Bayley, 2007). Previous neuroimaging studies on memory have primarily focused on stimulus evoked activity during the encoding or retrieval phases, and little attention has been paid to the neural processes that occur in the absence of direct external stimulation following learning. If neural connections are being strengthened during the post training consolidation period we would expect changes in connectivity, even at rest, between the brain regions involved in memory formation. One way to examine changes in resting-state connectivity during memory formation is to examine functional connectivity before and after a well understood form of learning such as Pavlovian fear conditioning.

In Pavlovian fear conditioning a previously neutral conditional stimulus (CS) is paired with an aversive unconditional stimulus (UCS). Through repeated pairings the CS can evoke a learned conditional response (CR) that is typically similar to the fearful unconditional response (UCR) that is evoked by the UCS (Pavlov, 1927). The amygdala is an area of convergence for the sensory signals of the CS and UCS, and neural plasticity in the amygdala is necessary for this type of learning (for review see Fanselow and Poulos, 2005; Kim and Jung, 2006; Helmstetter et al., 2008). Neuroimaging studies on fear conditioning have also focused on amygdala activity. Results from these studies have been largely consistent with work in laboratory animals providing further evidence that the amygdala is involved in this type of learning (Buchel et al., 1998; LaBar et al., 1998; Phelps and LeDoux, 2005).

Functional connectivity can be assessed using neuroimaging tools to understand changes in the fear circuit that occur during consolidation. Connectivity approaches can provide information about which brain regions share correlated activity. Functional connectivity can be measured in a resting-state. RSFC methods examine the spontaneous, low frequency (<0.1 Hz) fluctuations that occur in the absence of direct stimulation (Fox and Raichle, 2007; Biswal et al., 2010). Resting-state connectivity measures are relatively stable across time (Shehzad et al., 2009). However, differences in connectivity have been observed following behavioral tasks (Duff et al., 2008; Grigg and Grady, 2010; Stevens et al., 2010; Kim et al., 2011; Veer et al., 2011) or surgical procedures that result in behavioral changes (Pizoli et al., 2011). Changes in RSFC indicate that prior experience can modify neural networks at rest. These changes might also reflect some of the ongoing processes that support the consolidation of memory. The current study was designed to determine if the resting-state connectivity between the amygdala and other brain regions changes following fear conditioning. Furthermore, we wanted to identify whether or not any observed changes in connectivity were related to behavioral performance.

## Methods

### Participants

Thirty-two (15 women) right-handed, neurologically normal undergraduates from the University of Wisconsin-Milwaukee ranging from 18 to 37 years old (*M* = 22 years, SEM = 0.85) volunteered for the study. Three participants were excluded from analysis due to excessive head movement and two were excluded due to recording errors. The final sample included 27 participants. Participants were offered extra-credit in a psychology course, $20 and a picture of their brain. All participants supplied informed consent. All procedures were approved by the Institutional Review Boards for human subject research at the University of Wisconsin-Milwaukee and the Medical College of Wisconsin.

### Apparatus

#### Electrical stimulus

The UCS in the conditioning phase was a 500 ms duration electrical stimulation delivered via an AC (60 Hz) source (Contact Precision Instruments, Model SHK1, Boston, MA) through two surface cup electrodes (silver/silver chloride, 8 mm diameter, Biopac model EL258-RT, Goleta, CA). The electrodes were filled with electrolyte gel (Signa Gel, Parker Laboratories, Fairfield, NJ) and placed on the skin over the subject's right tibial nerve above the right medial malleous. Each participant determined the maximum UCS intensity used in the experiment individually prior to the start of the experiment in a work up procedure. The work up procedure consisted of no more than five presentations of the electrical stimulation. Each presentation was rated by the participant on a scale from 0 to 10 (0 = *no sensation*, 10 = *painful*, *but tolerable*). The intensity of the electrical stimulation was increased until the participant rated it as a 10. Participants were able to rate the stimulation higher than a 10 at which point the intensity would be decreased. The UCS intensity was set at the level that each participant rated as definitely painful, but tolerable (*M* = 3.56 mA, SEM = 0.28).

#### UCS expectancy

Participants manipulated a custom-made rotary dial to report their expectancy of receiving the UCS throughout the conditioning portion of the study. The dial controlled a cursor that was presented at the bottom of the visual display. Real-time feedback of the position of the cursor was continually presented. The dial was strapped around the participants' right thigh. Participants were instructed to manipulate the dial with their right hand. Participants received verbal instructions on how to use the dial before the experiment began. They were instructed to place the cursor at 0 if they were certain that they would not experience the UCS, at 50 if they were not sure if they would experience the UCS, and at 100 if they were certain that they would experience the UCS. Participants were instructed to update the position of the cursor continuously throughout the conditioning portion of the experiment. Participants were not instructed about any of the potential relationships between the visual stimuli and the UCS.

#### Skin conductance

Skin conductance was recorded using a Contact Precision Instruments unit (Boston, MA) with a SC5 24-bit digital amplifier from Contact Precision Instruments at 80 Hz. Psychlab software (London, UK) was used for skin conductance analysis. Skin conductance data was collected with electrodes (Biopac, Goleta, CA; Model EL258-RT) filled with electrolytic gel (Signagel, Parker Laboratories, Fairfield, NJ). The electrodes were attached to the sole of the left foot 2 cm apart.

#### Visual stimuli

The experiment was conducted using Presentation software (Albany, CA) on a Dell Inspiron 9300 (Round Rock, TX) laptop computer. Visual stimuli and the UCS expectancy rating bar were presented to participants while they were in the scanner using a back projection system with prism glasses mounted on the head coil. The visual stimuli were a green trapezoid and an orange pentagon. Assignment of the visual stimuli to be the CS+ and the CS− was counterbalanced.

#### MRI

Whole brain imaging was conducted using a 3T short bore Signa Excite MRI system. Functional images were collected using a T2^*^ weighted gradient-echo, echoplanar pulse sequence. We collected 4 mm sagittal slices (TR = 2 s; TE = 25 ms; field of view = 24 cm; flip angle = 90°; voxel size = 3.75 × 3.75 × 4.0 mm) during the experiment. The pre-conditioning and post-conditioning resting state runs consisted of 240 whole brain scans. The conditioning run consisted of 290 whole brain scans. High resolution spoiled gradient recalled (SPGR) images (1 mm slices) were collected in a sagittal orientation (TR = 9.6 s; TE = 3.9 ms; field of view = 24 cm; flip angle = 12°; voxel size = 0.9375 × 0.9375 × 1.0 mm) and served as an anatomical map for the functional images.

### Procedure

**Figure 1:**
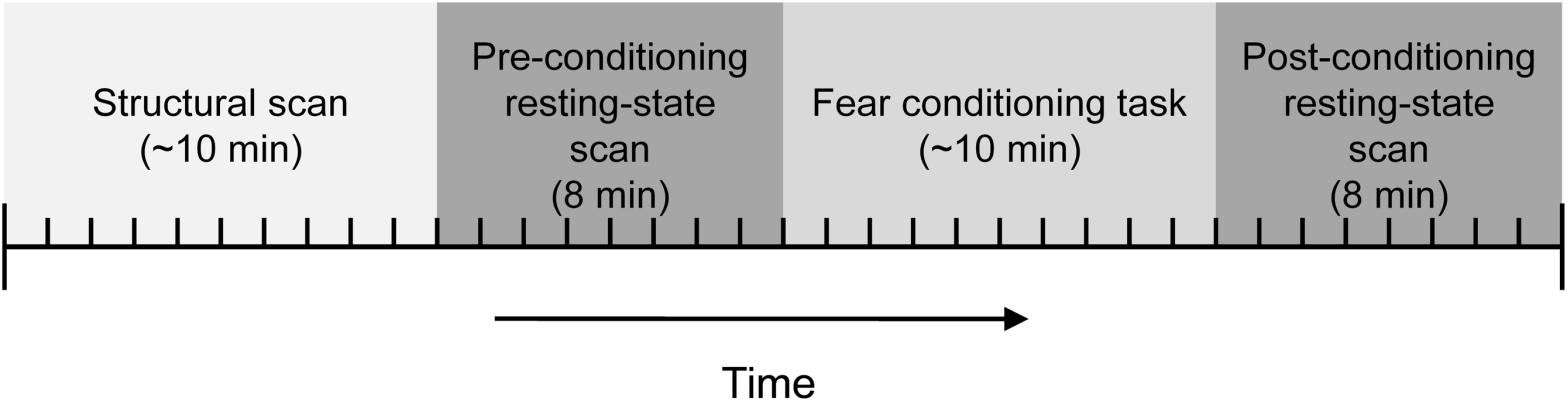
**Diagram of the phases in the experiment**.

Participants were instructed on how to use the UCS expectancy dial and the UCS intensity work up was completed prior to them entering the scanner. The high-resolution structural data was collected before the resting-state and conditioning task functional scans. A resting-state scan was collected next. Participants were instructed to keep their eyes closed during the resting-state scans. The conditioning task was next and consisted of 10 presentations of the CS+ and 10 presentations of the CS−. The duration of the CSs was 8 s. The CS+ always co-terminated with a 500 ms presentation of the UCS. The CSs were counterbalanced across subjects and presented in one of two pseudorandom trial order sequences with the caveat that there could be no more than two consecutive trials of the same type. A variable intertrial interval was used with a mean interval of 20 s. The basic paradigm is similar to several other imaging studies (Knight et al., 1999, 2004; Cheng et al., 2003). Another resting-state scan was collected following the conditioning task; see Figure 1.

### Data analysis

#### UCS expectancy

The UCS expectancy measure was defined as the mean expectancy rating during the last 4 s of the CS period on each trial for each participant. A CS type by trial ANOVA was calculated. An alpha value of 0.05 was used for all analyses unless specified otherwise.

#### Skin conductance response

SCR was analyzed by subtracting the mean of a 2 s baseline period prior to CS onset from the peak of the response during the entire CS presentation (Pineles et al., 2009) on each trial for each participant. A CS type by trial ANOVA was calculated.

#### General fMRI

Reconstruction and imaging processing was completed with AFNI (Cox, 1996). Raw data was motion corrected, passed through an edge detection algorithm and registered to the fifth volume of the functional run. The data were visually inspected for large head movements. Images containing large, discrete head movements were censored. Participants with excessive head movement (greater than 2.5 mm displacement or more than five examples of discrete head movement) were excluded from further analysis.

#### Conditioning fMRI

High-resolution structural scans were warped to Talairach space by manually placing anatomical markers consistent with previous studies (Balderston et al., 2011). We used the AFNI program 3dDeconvolve to model the mean impulse response function (IRF) evoked by the CS+ and the CS−. Head motion and motor processes associated with the gross movement of the UCS expectancy dial were included as regressors of no interest. Six images starting at stimulus onset were used to calculate the percent area under the curve (%AUC) of the mean IRF for each stimulus type. The resulting maps were blurred using a 4 mm full-width at half-maximum Gaussian kernel. These maps were used in the group analysis. Cluster thresholding (Forman et al., 1995) was used to correct for multiple comparisons across all the voxels in the whole brain volume (*p* = 10^−3^; rmm = 2; volume = 200 μL; corrected *p* < 0.05) with the use of Monte Carlo simulations in the AFNI program Alphasim. An additional region of interest (ROI) analysis was conducted on the amygdala. The Freesurfer software package was used for segmentation of the white matter, ventricles, and amygdala (Fischl et al., 2002, 2004). Freesurfer created volumes were realigned to native space using AFNI. The realigned volumes were then manually edited to conform to previously published standards (Morey et al., 2009). A CS type by laterality ANOVA was conducted on the %AUC data extracted from both the right and left amygdala ROIs.

#### Resting-state fMRI

Variability in the BOLD time series accounted for by respiration and cardiac rhythm was removed from the raw data using previously published methods (Birn et al., 2006) in which cardiac and respiratory signals and their first harmonics serve as variables in a multiple regression analysis. Baseline, drift, and head motion effects were removed from the time series using AFNI's 3dDeconvolve command. The mean signal from the Freesurfer created white matter and ventricle maps, as well as a global signal measure were also included as regressors of no interest as discussed by Fox and colleagues (2009). At this point the AFNI program 3dBandpass was used to apply a band pass filter to the time series to attenuate frequencies above 0.1 Hz and below 0.01 Hz. The amygdala time series was extracted from the individual Freesurfer generated amygdala masks for the left and right hemisphere for each participant. The mean amygdala time series, including the left and right hemisphere, for each participant was calculated and then correlated with the time series from every other voxel in the brain using AFNI's 3dDeconvolve command. The individual *r* statistics were then normalized using a Fisher's *z* transformation and resampled into 1 mm^3^ voxels. The normalized data was used to calculate all group level statistics. As a first pass analysis we calculated a whole-brain one-sample *t*-test on the normalized amygdala correlation coefficients for both the pre-conditioning and post-conditioning resting-state runs. Cluster thresholding was applied to the results (*p* = 10^−3^; rmm = 2; volume = 120 μL; corrected *p* < 0.05). A *t*-test directly comparing the pre-conditioning resting-state correlations values to the post-conditioning resting-state correlation values was also calculated. Cluster thresholding was also applied to the two-sample *t*-test results (*p* = 0.005; rmm = 2; volume = 120 μL; corrected *p* < 0.05).

To examine the relationship between behavioral performance and the change in amygdala connectivity from the pre-conditioning to post-conditioning resting-state scans we created a metric for both UCS expectancy and SCR performance. The UCS expectancy performance score was calculated by subtracting the mean CS− expectancy value from the CS+ expectancy value on a subject by subject basis. The SCR performance score was calculated by subtracting the mean CS− response from the mean CS+ response and dividing by the mean UCR value on a subject by subject basis. The change in amygdala connectivity was calculated by subtracting the normalized amygdala correlation coefficient from the pre-conditioning resting-state scan from the post-conditioning resting-state scan. We ran a step-wise multiple regression using the AFNI command 3dRegAna with UCS expectancy and SCR performance as regressors and the change in amygdala connectivity as the dependent variable. Cluster thresholding was applied to the results from the regression analysis (*p* = 0.005; rmm = 2; volume = 120 μL; corrected *p* < 0.05).

## Results

### Behavioral data

**Figure 2:**
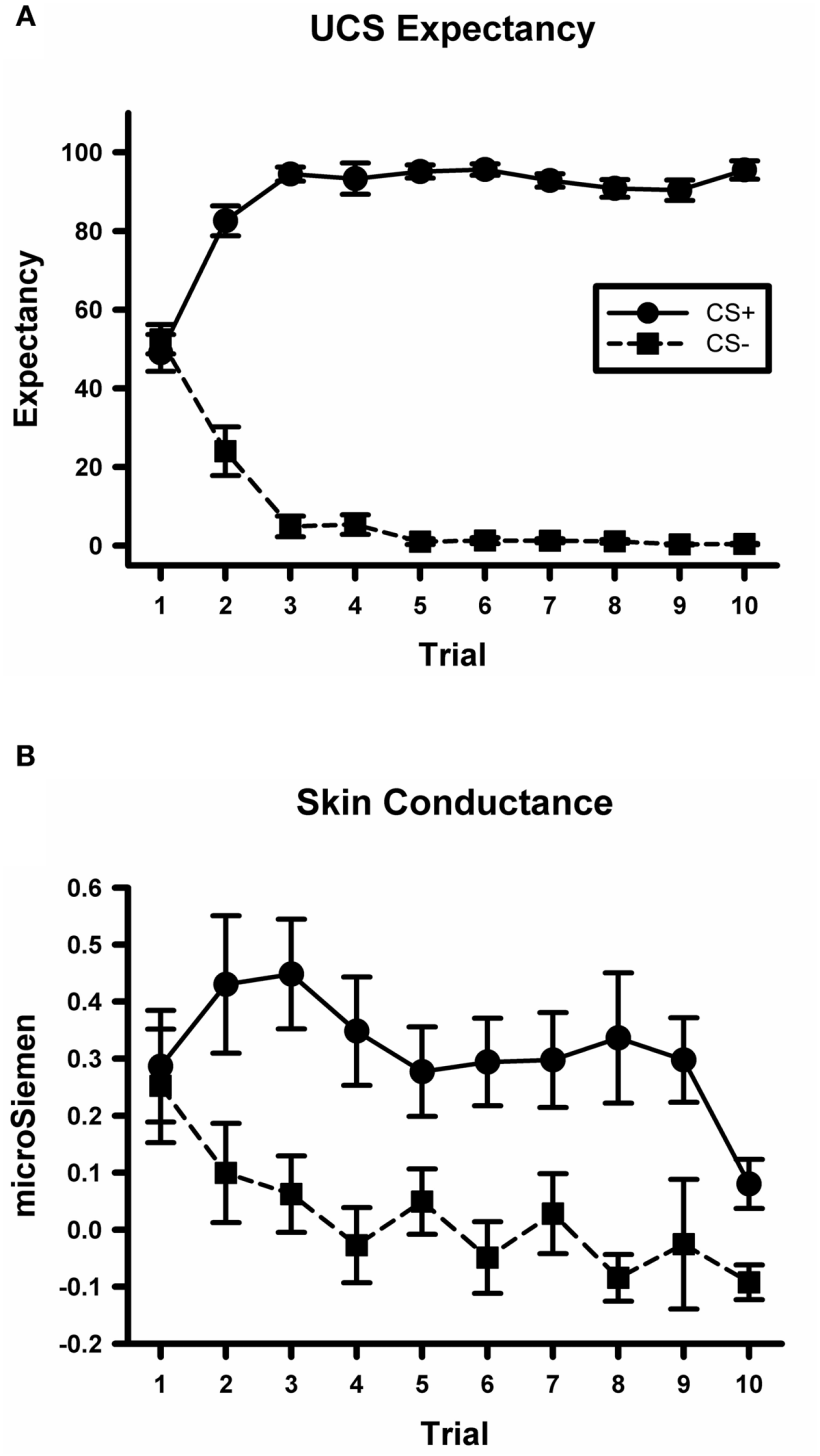
**Participants demonstrate differential conditioning with UCS expectancy ratings and SCR. (A)** Mean UCS expectancy ratings on CS+ and CS− trials during the conditioning task. **(B)** Mean SCR on CS+ and CS− trials during the conditioning task.

A CS type by trial ANOVA conducted on the UCS expectancy data yielded a significant main effect for CS type, *F*_(1, 234)_ = 2373.8, *p* < 0.001. There was also a significant main effect for trial, *F*_(9, 234)_ = 2.6, *p* < 0.01. The CS type by trial interaction was also significant, *F*_(9, 234)_ = 69.5, *p* < 0.001; see Figure 2A. Simple effects tests did not detect any difference between UCS expectancy on the first CS+ and CS− trial, *F*_(1, 234)_ < 1. However, there was a significant difference by the last trial, *F*_(1, 234)_ = 684, *p* < 0.001, with higher expectancy ratings on the CS+ trial than on the CS− trial. These data indicate that participants did not differentially expect the UCS on early trials. Through the course of acquisition they learned to expect the UCS to occur on CS+ trials and not to expect the UCS on CS− trials.

A CS type by trial ANOVA conducted on the skin conductance data yielded a significant main effect for CS type, *F*_(1, 234)_ = 25.6, *p* < 0.001; see Figure 2B. There was not a significant main effect for trial or for the CS type by trial interaction, largest *F* = 1.8, *p* = 0.07. These data indicate that participants exhibited larger SCRs on CS+ trials than on CS− trials which is consistent with previous studies of fear acquisition from our lab (Knight et al., 2004; Cheng et al., 2007).

### Conditioning fMRI data

**Figure 3:**
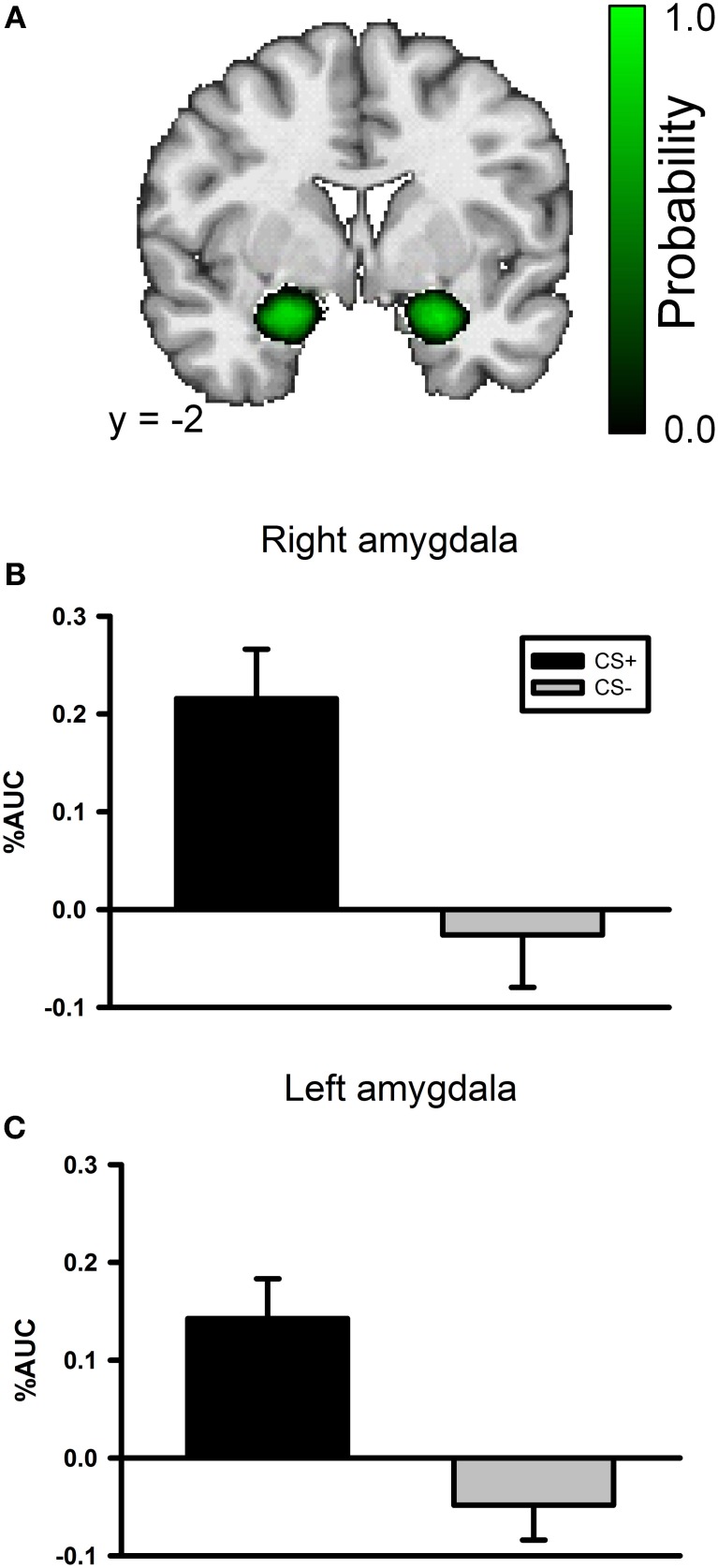
**The left and right amygdala both demonstrate differential responses to the CSs in the conditioning phase of the experiment. (A)** An amygdala probability mask created by combining each participant's anatomical amygdala ROI. The color scale corresponds to the probability that the region is included in any of the participants' amygdala ROIs. Black indicates a low probability and bright green indicates high probability. **(B)** Bar graph depicting the AUC values for both the CS+ and CS− evoked BOLD response in the right amygdala during the conditioning task. **(C)** Bar graph depicting the AUC values for both the CS+ and CS− evoked BOLD response in the left amygdala during the conditioning task.

A whole brain ANOVA contrast between the CS+ and CS− evoked response identified a variety of brain regions that have been previously implicated in fear conditioning including the insula and visual cortex; see Table 1. However, we did not observe a significant conditioning effect in the amygdala with the whole brain ANOVA. The lack of an amygdala effect in a whole brain analysis is not unique. Several studies have not identified amygdala activation using a whole brain analysis approach (Knight et al., 1999; Phelps et al., 2004) or have observed a transient amygdala effect which is evident early in acquisition, but diminishes over time (Buchel et al., 1998; LaBar et al., 1998). We conducted a ROI analysis of the amygdala due to our strong *a priori* prediction that it would be involved in this task. The amygdala ROI mask was created for each participant using Freesurfer; see Figure 3A. A CS type by laterality ANOVA on the CS evoked responses in the amygdala identified a significant main effect for CS type, *F*_(3, 104)_ = 23.2, *p* < 0.001, but no significant effect for laterality and no significant CS type by laterality interaction, largest *F* = 1.13, *p* = 0.291; see Figures 3B,C.

**Table 1:** **Brain activation during fear conditioning**.

**Region**	**Hemisphere**	**Volume (mm^3^)**	**Talairach coordinates**		
			**RL**	**AP**	**IS**
Thalamus/Caudate	Bilateral	12646	0	−23	12
Lingual Gyrus/Culmen/Cuneus	Right	3985	6	−58	0
Superior Frontal Gyrus/Precentral Gyrus	Left	3298	−20	−12	69
Superior Frontal Gyrus/Cingulate Gyrus	Right	1895	1	4	48
Precuneus	Right	1788	2	−55	63
Postcentral Gyrus/Insula	Left	995	−60	−19	21
Lentiform Nucleus/Putamen	Left	690	−20	0	−7
Postcentral Gyrus	Left	639	−45	−31	57
Superior Temporal Gyrus/Inferior Frontal Gyrus/lnsula	Left	560	−49	11	1
Middle Frontal Gyrus	Right	483	34	−7	60
Inferior Frontal Gyrus	Right	392	50	19	−5

*CS+ > CS− for all clusters*.

### Functional connectivity data

**Figure 4:**
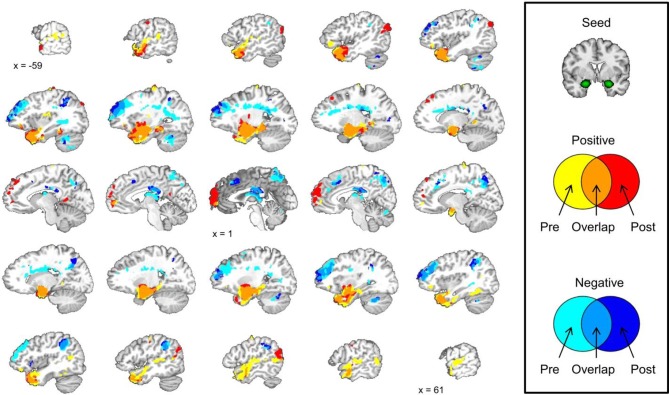
**Amygdala functional connectivity before and after the conditioning task shows some similarities and some differences.** Brain images depict the pre- and post-conditioning connectivity maps as well as where those maps overlap.

We did not observe any laterality effects in the amygdala data from the conditioning session so we correlated the mean amygdala signal during the pre-conditioning resting-state scan with the signal from all of the voxels in the brain during the same scan. The same analysis was conducted with the mean amygdala signal during the post-conditioning resting-state scan. A correlation map with the mean amygdala signal was created for the pre-conditioning resting-state scan and the post-conditioning resting-state scan. These maps were layered on top of one another creating a map that displays areas that show significant positive and negative correlations with the mean amygdala signal during pre- and post-conditioning scans. This map also displays regions that are significantly positively or negatively correlated with the amygdala at both resting-state scans and regions that changed from a positive to negative correlation from pre- to post-conditioning or vice versa; see Figure 4.

### Amygdala connectivity is stable across time

We examined amygdala connectivity at two time points, once prior to conditioning task, and once following conditioning. The second resting-state scan began approximately 12 min after the completion of the first resting-state scan. Amygdala activity was positively correlated with activity in the medial temporal lobe and ventral portions of the prefrontal cortex (PFC) before and after the conditioning task. Amygdala activity was negatively correlated with activity in the dorsal lateral PFC, precuneus, posterior cingulate cortex, and an area of parietal cortex near the junction of the inferior parietal lobule and the supramarginal gyrus before and after the conditioning task. These results are consistent with previous studies that have examined amygdala functional connectivity at one time point at rest (Roy et al., 2009; Kim et al., 2011).

### Amygdala connectivity is altered following conditioning

Amygdala connectivity is relatively consistent before and after the conditioning task, but some the connectivity of some regions is different following conditioning. The medial PFC shows a significant positive correlation with amygdala activity following the conditioning task that was not present prior to conditioning. The left angular gyrus and right posterior temporal lobe show a similar pattern with a significant positive correlation with the amygdala after the conditioning task that was not present prior to conditioning.

**Figure 5:**
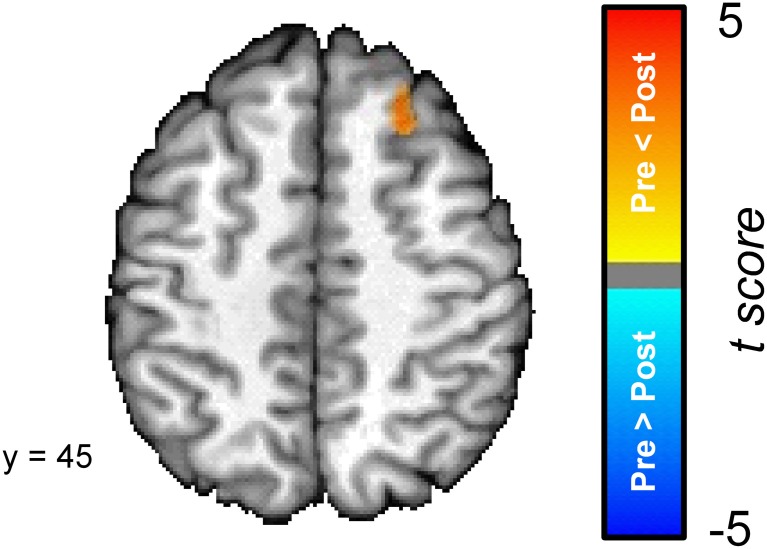
**The dorsal medial prefrontal cortex (superior frontal gyrus) [Talairach coordinates: 24, 30, 45] shows a significant increase in its connectivity with the amygdala following the conditioning task.** The colors on the brain map correspond to the *t*-values on the color scale.

In order to explore the differences between the pre- and post-conditioning amygdala connectivity maps we directly compared the two; see Figure 5. A cluster in the superior frontal gyrus was the only region that was characterized by a significant difference in connectivity between the pre- and post-conditioning resting-state scans. The superior frontal gyrus cluster showed a significant increase in connectivity between the two scans.

### Changes in amygdala connectivity correlate with learning and behavioral performance

**Figure 6:**
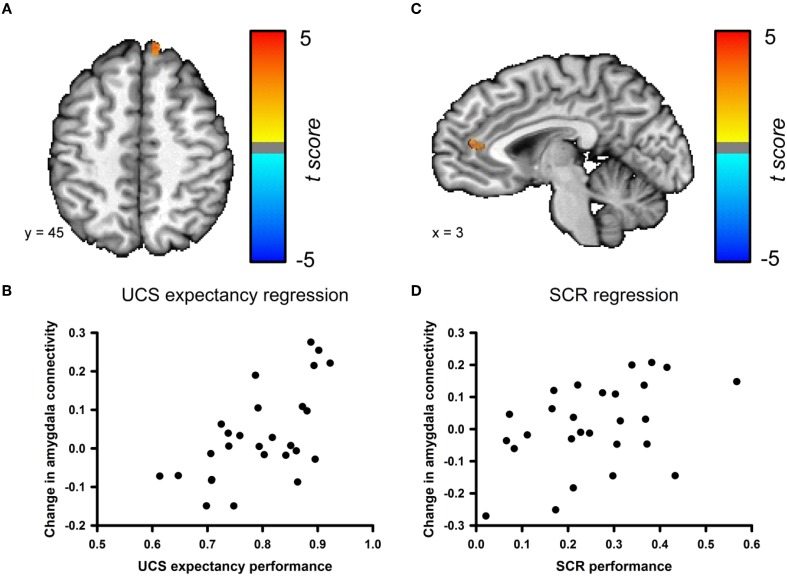
**Changes in amygdala connectivity are related to behavioral performance. (A)** Connectivity changes between the amygdala and the superior frontal gyrus [Talairach coordinates: 11, 48, 9] are positively correlated with UCS expectancy performance during the conditioning task. **(B)** Scatter plot depicting UCS expectancy performance and change in connectivity between the amygdala and superior frontal gyrus. **(C)** Connectivity changes between the amygdala and the ACC [Talairach coordinates: 3, 13, −5] are positively correlated with SCR performance during the conditioning task. **(D)** Scatter plot depicting SCR performance and change in connectivity between the amygdala and the ACC.

We examined the relationship between changes in amygdala connectivity before and after conditioning and behavioral performance using a step-wise multiple regression analysis. UCS expectancy was the first regressor entered into the model. UCS expectancy performance positively correlated with the change in amygdala connectivity for a cluster in the superior frontal gyrus; see Figures 6A,B. This cluster neighbors the superior frontal gyrus cluster that was identified in the pre- versus post-conditioning amygdala connectivity contrast. The second step of the regression analysis examined if any of the residual variance in the change in amygdala connectivity could be accounted for by SCR performance. SCR performance positively correlated with the change in amygdala connectivity for a cluster in the anterior cingulate cortex (ACC); see Figures 6C,D. The changes in connectivity that correlate with behavioral performance suggest that this phenomenon is due to associative learning. In order to ensure that the changes in connectivity were not due participants having a higher level of anxiety in the resting-state scan that followed conditioning, we examined the heart rate data from both of the resting-state scans. We found that the average heart rate before conditioning (*M* = 67.32, SEM = 2.85) was not significantly different from the average heart rate after the conditioning session (*M* = 70.05, SEM = 2.98), *t*_(52)_ = −0.663, *p* = 0.51 (data not shown).

## Discussion

This study examined changes in the functional connectivity of the amygdala before and after subjects encoded a new fear conditioning memory. Behavioral results indicate that participants learned normally as measured by UCS expectancy and SCR (Cheng et al., 2007; Schultz and Helmstetter, 2010). fMRI data from the conditioning session revealed a network of regions that showed differential responses to the CS+ and the CS− that are consistent with previous studies on fear conditioning (Buchel et al., 1998; LaBar et al., 1998; Knight et al., 1999). RSFC analyses revealed that activity in the amygdala was positively correlated with activity in the medial temporal lobe and ventral PFC before and after the completion of the conditioning task. Activity in the amygdala was negatively correlated with activity in the dorsal lateral PFC, precuneus, posterior cingulate cortex, inferior parietal lobule, and the supramarginal gyrus before and after conditioning. While some regions demonstrated consistent functional connectivity with the amygdala across the scans, other regions were characterized by differences in amygdala connectivity before and after conditioning. A direct comparison between pre- and post-conditioning connectivity maps revealed a cluster in the superior frontal gyrus that significantly increased in connectivity with the amygdala following conditioning. Furthermore, we found that changes in amygdala connectivity from the pre-conditioning to the post-conditioning resting scans were correlated to behavioral performance. UCS expectancy performance positively correlated with the change in connectivity in the superior frontal gyrus and SCR performance positively correlated with the change in connectivity between the amygdala and the ACC.

We found that participants acquired differential fear measured by UCS expectancy ratings and by SCR. The fMRI results from the conditioning session identified several brain regions that exhibited a larger BOLD response to the CS+ than to the CS−. The thalamus and caudate were part of a large cluster that was characterized by differential CS+ and CS− responses. This finding is consistent a previous study in a normal population (Knight et al., 2004), as well as a study that examined differential fear in patients with specific phobias (Schweckendiek et al., 2011). The insula also exhibited differential BOLD responses in the current study. A previous study also found differential insula activation when participants were aware of the stimulus contingencies (Tabbert et al., 2011). Differential CS evoked responses were also found in visual cortex. Visual cortex activity has been reported in a variety of other studies that have used visual CSs (Knight et al., 1999; Morris et al., 2001; Cheng et al., 2003; Tabbert et al., 2005). We also found differential CS evoked activity in the amygdala with an anatomical ROI analysis which is consistent with its role in fear conditioning (Fanselow and Poulos, 2005; Kim and Jung, 2006). Overall, the fMRI results from the conditioning session in the current study are very consistent with results from previous published studies using similar parameters.

A direct comparison of the pre- and post-conditioning resting-state amygdala correlation maps revealed a cluster in the superior frontal gyrus that was significantly more positively correlated with the amygdala after conditioning than it was before. This increase in connectivity could reflect the process of consolidating the memory of the CS-UCS contingency and the underlying strengthening of neural connections that support the permanent storage of this memory. Several studies suggest that network level changes occur in order to support a memory after the initial learning event (for review see Frankland and Bontempi, 2005). Examining resting-state changes following conditioning is a convenient way to explore network level changes because participants can undergo a resting-state scan at various points following the task and connectivity can be assessed at several time points without the need to present the conditioning stimuli and confound the results with retrieval and extinction effects.

A recent study by Kim and colleagues (2011) also examined amygdala resting-state connectivity. They found a positive correlation between baseline anxiety scores and functional connectivity between the amygdala and a more medial portion of the dorsal PFC. Participants with higher anxiety scores showed a stronger positive correlation between the amygdala and dorsal PFC. Unfortunately, we did not collect anxiety scores and we cannot rule out the possibility that the conditioning task increased anxiety which could also lead to increased amygdala-dorsal PFC connectivity. However, another study measured amygdala functional connectivity after either a social stress or control manipulation (Veer et al., 2011) and found that the stress manipulation increased connectivity between the amygdala and ventral PFC. The authors suggested that this difference in connectivity could be attributed to a homeostatic process in which the ventral PFC is involved in the stress recovery process. If the results of the current study were simply due to the induction of anxiety or stress state we would have likely observed increased connectivity between the amygdala and ventral PFC as a reflection of recovery from this state.

The amygdala connectivity changes observed in the superior frontal gyrus in the current study might reflect network level changes that occur to support the formation of memory. This finding is consistent with a study that found that a similar region of superior frontal gyrus was activated when participants recalled multimodal associations that had been learned 24 h earlier (Stock et al., 2009). The superior frontal gyrus was also activated in a study where participants recalled long-term episodic memories (Viard et al., 2007). The superior frontal gyrus appears to be an important brain region involved in long-term memory storage. The changes in amygdala connectivity with the superior frontal gyrus in the current study may reflect the ongoing process of strengthening synapses between the amygdala which is critical for the acquisition of fear conditioning and the superior frontal gyrus which is important for the long-term storage of this memory.

Connectivity changes between the superior frontal gyrus and the amygdala were also related to behavioral performance. Participants with better UCS expectancy performance showed a larger increase in connectivity between these regions. UCS expectancy is an explicit measure of learning and these connectivity changes may reflect the consolidation of this type of memory. SCR performance was related to changes in connectivity between the ACC and the amygdala. Participants with better SCR performance showed a larger increase in connectivity between the amygdala and anterior cingulate. Milad and colleagues (2007) identified a relationship between cortical thickness in the dorsal ACC and SCR performance. Another recent study (Linnman et al., 2012) found that resting metabolism in the dorsal ACC was positively correlated with SCR performance. The findings from the current study are consistent with the ACC having a role in SCR performance. We demonstrate for the first time that changes in connectivity between the ACC and amygdala are related to SCR performance in addition to cortical thickness and resting metabolism in this area. Changes in amygdala connectivity and performance on our two behavioral measures were related, but different patterns emerged for the explicit measure, UCS expectancy, and the implicit measure, SCR. This is consistent with the idea that explicit and implicit measures of learning are dissociable (Schultz and Helmstetter, 2010) and that these two different types of memory are supported by different neural circuits (Knight et al., 2009).

We observed changes in amygdala resting-state connectivity following fear conditioning. However, it is important to note that these changes could be influenced by a variety of factors. Future studies should focus on eliminating the possibility that connectivity changes are due to non-associative factors. Exposure to fearful visual stimuli is not a likely explanation for the results of the current study as Kim and colleagues (2011) found no differences in amygdala connectivity at rest between one group that was exposed to images of fearful faces before the resting-state scan and another group that saw the same faces after the resting-state scan. It will be important for future studies to include a control group that is exposed to the same stimuli, but do not learn a specific CS-UCS association. The current study did not include that type of control group. However, the correlations between behavioral measures of learning and changes in amygdala connectivity strongly suggest that the observed changes in functional connectivity are specifically related to this type of learning. This study is an important step in understanding the network level changes that occur to support the offline processing and storage of this type of memory. A better understanding of this process might open the door for new and improved clinical interventions for anxiety and fear related disorders.

### Conflict of interest statement

The authors declare that the research was conducted in the absence of any commercial or financial relationships that could be construed as a potential conflict of interest.
